# 2-(2,4-Dichloro­phen­oxy­meth­yl)-5-(4-methyl­phen­yl)imidazo[2,1-*b*][1,3,4]thia­diazole^1^

**DOI:** 10.1107/S160053681300384X

**Published:** 2013-02-13

**Authors:** Bakr F. Abdel-Wahab, Hanan A. Mohamed, Seik Weng Ng, Edward R. T. Tiekink

**Affiliations:** a Applied Organic Chemistry Department, National Research Centre, Dokki, 12622 Giza, Egypt; b Department of Chemistry, University of Malaya, 50603 Kuala Lumpur, Malaysia; c Chemistry Department, Faculty of Science, King Abdulaziz University, PO Box 80203 Jeddah, Saudi Arabia

## Abstract

In the title compound, C_18_H_13_Cl_2_N_3_OS, the eight atoms comprising the central imidazo/thia­diazo­lethia­diazole residue are coplanar (r.m.s. deviation = 0.009 Å). The dihedral angle of 8.72 (13)° between the dichloro­benzene and tolyl rings reflects a twist about the O—C(benzene) bond; the C_m_—O—C_b_—C_b_ torsion angle = −168.5 (2)° (m = methyl­ene C and b is benzene C). Supra­molecular tapes along the *b* axis are found in the crystal structure which are mediated by π–π inter­actions occurring between centrosymmetrically related thia­diazole rings [inter-ring centroid distance = 3.6907 (16) Å] and between the benzene and tolyl rings [inter-ring centroid distance = 3.7597 (16) Å].

## Related literature

For background to the biological activity of imidazothia­dia­zo­les, see: Abdel-Wahab *et al.* (2011 ▶); Karki *et al.* (2011 ▶); Khazi *et al.* (2011 ▶). For the synthesis, see: Abdel-Wahab *et al.* (2011 ▶). For a related structure, see: Fun *et al.* (2011 ▶).
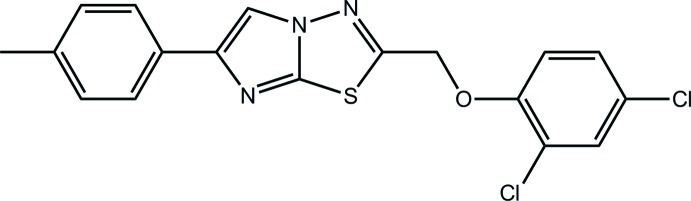


## Experimental

### 

#### Crystal data

C_18_H_13_Cl_2_N_3_OS
*M* *_r_* = 390.27
Triclinic, 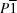

*a* = 8.3015 (7) Å
*b* = 8.3053 (7) Å
*c* = 14.4374 (13) Å
α = 97.180 (7)°
β = 92.644 (7)°
γ = 118.996 (9)°
*V* = 857.25 (13) Å^3^
*Z* = 2
Mo *K*α radiation
μ = 0.51 mm^−1^
*T* = 295 K
0.40 × 0.30 × 0.20 mm

#### Data collection

Agilent SuperNova Dual diffractometer with an Atlas detector
Absorption correction: multi-scan (*CrysAlis PRO*; Agilent, 2011 ▶) *T*_min_ = 0.856, *T*_max_ = 1.000
8391 measured reflections
3932 independent reflections
2659 reflections with *I* > 2σ(*I*)
*R*_int_ = 0.028

#### Refinement

*R*[*F* ^2^ > 2σ(*F* ^2^)] = 0.048
*wR*(*F* ^2^) = 0.172
*S* = 0.98
3932 reflections
227 parameters
H-atom parameters constrained
Δρ_max_ = 0.27 e Å^−3^
Δρ_min_ = −0.24 e Å^−3^

### 

Data collection: *CrysAlis PRO* (Agilent, 2011 ▶); cell refinement: *CrysAlis PRO*; data reduction: *CrysAlis PRO*; program(s) used to solve structure: *SHELXS97* (Sheldrick, 2008 ▶); program(s) used to refine structure: *SHELXL97* (Sheldrick, 2008 ▶); molecular graphics: *ORTEP-3 for Windows* (Farrugia, 2012 ▶) and *DIAMOND* (Brandenburg, 2006 ▶); software used to prepare material for publication: *publCIF* (Westrip, 2010 ▶).

## Supplementary Material

Crystal structure: contains datablock(s) global, I. DOI: 10.1107/S160053681300384X/hg5290sup1.cif

Structure factors: contains datablock(s) I. DOI: 10.1107/S160053681300384X/hg5290Isup2.hkl

Additional supplementary materials:  crystallographic information; 3D view; checkCIF report

## Figures and Tables

**Fig. 1.**
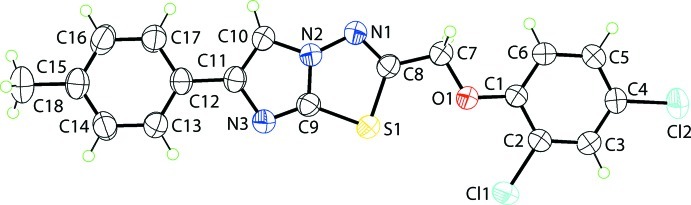
The molecular structure of (I) showing the atom-labelling scheme and displacement ellipsoids at the 50% probability level.

**Fig. 2.**
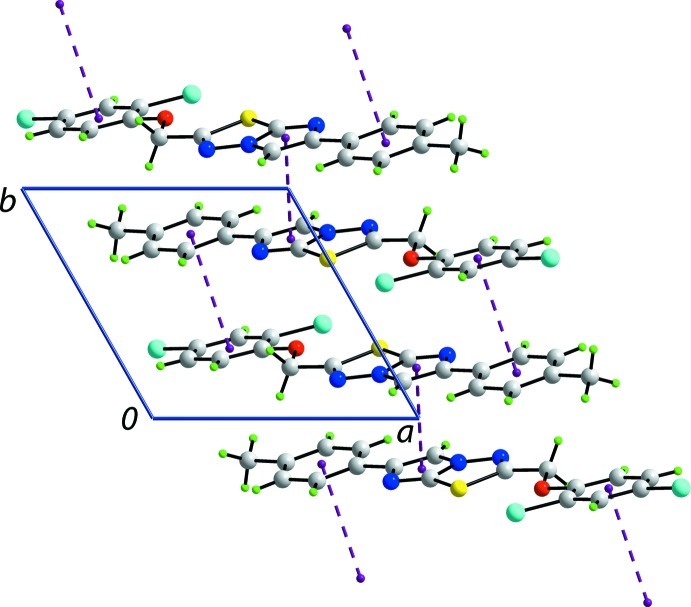
A view of the supramolecular tape along the b axis in (I) mediated by π—π interactions shown as purple dashed lines.

**Fig. 3.**
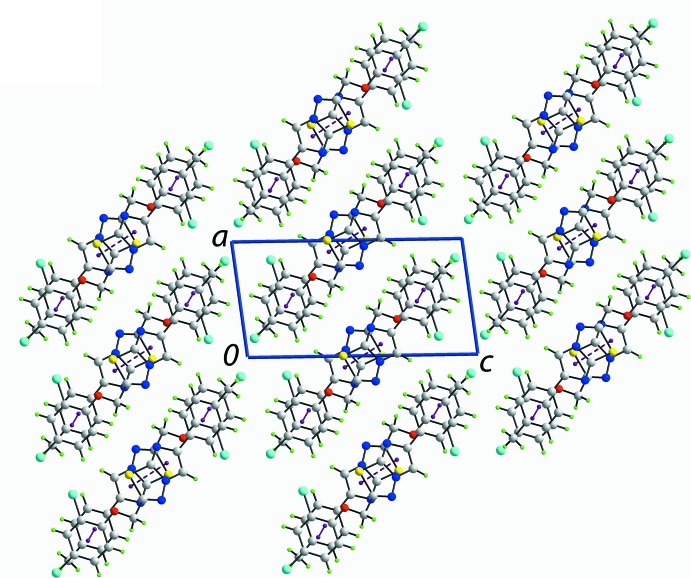
A view of the crystal packing in projection down the b axis. The π—π interactions are shown as purple dashed lines.

## supplementary crystallographic information

### Comment

The title compound (I) was investigated in relation to the established biological activities exhibited by imidazothiadiazoles (Abdel-Wahab *et al.*, 2011; Karki *et al.*, 2011; Khazi *et al.*, 2011).

In (I), Fig. 1, the eight atoms comprising the fused imidazo/thiadiazolethiadiazole residue are co-planar (r.m.s. deviation = 0.009 Å). This system forms dihedral angles of 6.01 (10) and 3.28 (11)° with the attached dichlorobenzene and tolyl rings, respectively. The r.m.s. deviation from the least-squares plane for all 25 non-hydrogen atoms is 0.085 Å with maximum deviations of 0.180 (2) and -0.197 (1) for the O1 and Cl1 atoms, respectively. This is consistent with a small twist about the C1—O1 bond with the C7—O1—C1—C2 torsion angle being -168.5 (2)°. The S and O atoms are *syn* and are separated by 2.823 (3) Å. A small twist was also observed in the structure of the closely related compound 2-isobutyl-6-phenylimidazo[2,1-*b*][1,3,4]thiadiazole (Fun *et al.*, 2011).

In the crystal packing, molecules aggregate into tapes along the *b* axis *via*π–π interactions occurring between centrosymmetrically related thiadiazole rings [inter-ring centroid distance = 3.6907 (16) Å for symmetry operation: 2 - *x*, -*y*, 1 - *z*] and between the benzene and tolyl rings [inter-ring centroid distance = 3.7597 (16) Å for symmetry operation: 2 - *x*, 1 - *y*, 1 - *z*], Fig. 2. Columns stack with no specific interactions between them, Fig. 3.

### Experimental

The title compound was prepared according to the reported method (Abdel-Wahab *et al.*, 2011). Colourless crystals were obtained from DMF solution by slow evaporation at room temperature.

### Refinement

Carbon-bound H-atoms were placed in calculated positions (C—H 0.93 to 0.97 Å) and were included in the refinement in the riding model approximation, with *U*_iso_(H) = 1.2*U*_equiv_(C) or 1.5*U*_equiv_(C).

### Crystal data

**Table:**

C_18_H_13_Cl_2_N_3_OS	*Z* = 2
*M**_r_* = 390.27	*F*(000) = 400
Triclinic, *P*1	*D*_x_ = 1.512 Mg m^−^^3^
Hall symbol: -P 1	Mo *K*α radiation, λ = 0.71073 Å
*a* = 8.3015 (7) Å	Cell parameters from 1915 reflections
*b* = 8.3053 (7) Å	θ = 2.8–27.5°
*c* = 14.4374 (13) Å	µ = 0.51 mm^−^^1^
α = 97.180 (7)°	*T* = 295 K
β = 92.644 (7)°	Prism, colourless
γ = 118.996 (9)°	0.40 × 0.30 × 0.20 mm
*V* = 857.25 (13) Å^3^	

### Data collection

**Table:**

Agilent SuperNova Dual diffractometer with an Atlas detector	3932 independent reflections
Radiation source: SuperNova (Mo) X-ray Source	2659 reflections with *I* > 2σ(*I*)
Mirror monochromator	*R*_int_ = 0.028
Detector resolution: 10.4041 pixels mm^-1^	θ_max_ = 27.5°, θ_min_ = 2.8°
ω scan	*h* = −10→10
Absorption correction: multi-scan (*CrysAlis PRO*; Agilent, 2011)	*k* = −10→10
*T*_min_ = 0.856, *T*_max_ = 1.000	*l* = −18→15
8391 measured reflections	

### Refinement

**Table:**

Refinement on *F*^2^	Primary atom site location: structure-invariant direct methods
Least-squares matrix: full	Secondary atom site location: difference Fourier map
*R*[*F*^2^ > 2σ(*F*^2^)] = 0.048	Hydrogen site location: inferred from neighbouring sites
*wR*(*F*^2^) = 0.172	H-atom parameters constrained
*S* = 0.98	*w* = 1/[σ^2^(*F*_o_^2^) + (0.1*P*)^2^] where *P* = (*F*_o_^2^ + 2*F*_c_^2^)/3
3932 reflections	(Δ/σ)_max_ = 0.001
227 parameters	Δρ_max_ = 0.27 e Å^−^^3^
0 restraints	Δρ_min_ = −0.24 e Å^−^^3^

### Special details

**Table:**

Geometry. All e.s.d.'s (except the e.s.d. in the dihedral angle between two l.s. planes) are estimated using the full covariance matrix. The cell e.s.d.'s are taken into account individually in the estimation of e.s.d.'s in distances, angles and torsion angles; correlations between e.s.d.'s in cell parameters are only used when they are defined by crystal symmetry. An approximate (isotropic) treatment of cell e.s.d.'s is used for estimating e.s.d.'s involving l.s. planes.
Refinement. Refinement of *F*^2^ against ALL reflections. The weighted *R*-factor *wR* and goodness of fit *S* are based on *F*^2^, conventional *R*-factors *R* are based on *F*, with *F* set to zero for negative *F*^2^. The threshold expression of *F*^2^ > σ(*F*^2^) is used only for calculating *R*-factors(gt) *etc*. and is not relevant to the choice of reflections for refinement. *R*-factors based on *F*^2^ are statistically about twice as large as those based on *F*, and *R*- factors based on ALL data will be even larger.

### Fractional atomic coordinates and isotropic or equivalent isotropic displacement parameters (Å^2^)

**Table:**

	*x*	*y*	*z*	*U*_iso_*/*U*_eq_	
Cl1	0.83905 (10)	0.40968 (12)	0.15939 (6)	0.0701 (3)	
Cl2	0.16453 (12)	0.30193 (13)	0.04111 (6)	0.0730 (3)	
S1	1.00105 (10)	0.30491 (9)	0.41463 (5)	0.0493 (2)	
O1	0.6883 (3)	0.3049 (3)	0.32904 (14)	0.0540 (5)	
C1	0.5593 (3)	0.3020 (3)	0.26664 (19)	0.0429 (6)	
C2	0.6151 (4)	0.3510 (3)	0.1799 (2)	0.0462 (6)	
C3	0.4962 (4)	0.3525 (3)	0.1110 (2)	0.0494 (6)	
H3	0.5354	0.3847	0.0534	0.059*	
C4	0.3174 (4)	0.3053 (3)	0.12837 (19)	0.0476 (6)	
C5	0.2607 (4)	0.2588 (4)	0.2137 (2)	0.0522 (7)	
H5	0.1408	0.2286	0.2252	0.063*	
C6	0.3797 (4)	0.2565 (4)	0.2827 (2)	0.0497 (6)	
H6	0.3396	0.2244	0.3402	0.060*	
C7	0.6268 (4)	0.2235 (4)	0.40983 (19)	0.0492 (6)	
H7A	0.5252	0.0964	0.3920	0.059*	
H7B	0.5844	0.2946	0.4497	0.059*	
C8	0.7883 (4)	0.2255 (3)	0.46024 (18)	0.0431 (6)	
C9	1.0864 (4)	0.2605 (3)	0.51522 (18)	0.0444 (6)	
C10	1.0183 (4)	0.1610 (4)	0.6497 (2)	0.0506 (6)	
H10	0.9573	0.1154	0.7009	0.061*	
C11	1.1978 (4)	0.2117 (3)	0.63753 (18)	0.0444 (6)	
C12	1.3346 (4)	0.2063 (3)	0.70303 (19)	0.0455 (6)	
C13	1.5166 (4)	0.2710 (4)	0.6864 (2)	0.0538 (7)	
H13	1.5549	0.3204	0.6319	0.065*	
C14	1.6426 (4)	0.2638 (4)	0.7489 (2)	0.0584 (7)	
H14	1.7644	0.3099	0.7358	0.070*	
C15	1.5936 (4)	0.1901 (4)	0.8307 (2)	0.0541 (7)	
C16	1.4112 (5)	0.1263 (4)	0.8480 (2)	0.0650 (8)	
H16	1.3732	0.0769	0.9025	0.078*	
C17	1.2846 (4)	0.1347 (4)	0.7856 (2)	0.0607 (8)	
H17	1.1635	0.0915	0.7993	0.073*	
C18	1.7309 (5)	0.1820 (4)	0.8983 (2)	0.0712 (9)	
H18A	1.8126	0.1532	0.8640	0.107*	
H18B	1.6661	0.0869	0.9355	0.107*	
H18C	1.8019	0.3006	0.9389	0.107*	
N1	0.7783 (3)	0.1707 (3)	0.54018 (17)	0.0506 (5)	
N2	0.9469 (3)	0.1916 (3)	0.57041 (15)	0.0448 (5)	
N3	1.2402 (3)	0.2741 (3)	0.55163 (15)	0.0483 (5)	

### Atomic displacement parameters (Å^2^)

**Table:**

	*U*^11^	*U*^22^	*U*^33^	*U*^12^	*U*^13^	*U*^23^
Cl1	0.0431 (4)	0.1083 (6)	0.0602 (5)	0.0340 (4)	0.0157 (4)	0.0301 (4)
Cl2	0.0630 (5)	0.1060 (6)	0.0645 (5)	0.0519 (5)	−0.0016 (4)	0.0230 (5)
S1	0.0468 (4)	0.0648 (4)	0.0379 (4)	0.0278 (3)	0.0067 (3)	0.0129 (3)
O1	0.0417 (10)	0.0749 (11)	0.0495 (12)	0.0280 (9)	0.0083 (9)	0.0273 (9)
C1	0.0371 (13)	0.0467 (12)	0.0439 (15)	0.0189 (11)	0.0054 (11)	0.0120 (11)
C2	0.0374 (14)	0.0519 (13)	0.0486 (16)	0.0202 (12)	0.0068 (12)	0.0136 (12)
C3	0.0496 (16)	0.0583 (15)	0.0438 (16)	0.0277 (14)	0.0094 (13)	0.0153 (12)
C4	0.0454 (15)	0.0534 (13)	0.0480 (16)	0.0273 (12)	0.0015 (12)	0.0108 (12)
C5	0.0411 (15)	0.0637 (15)	0.0578 (18)	0.0289 (13)	0.0095 (14)	0.0165 (14)
C6	0.0447 (15)	0.0623 (15)	0.0467 (16)	0.0275 (13)	0.0112 (13)	0.0174 (13)
C7	0.0462 (15)	0.0559 (14)	0.0464 (16)	0.0244 (13)	0.0061 (13)	0.0155 (12)
C8	0.0450 (14)	0.0421 (12)	0.0401 (15)	0.0201 (11)	0.0035 (12)	0.0070 (11)
C9	0.0441 (14)	0.0473 (13)	0.0389 (15)	0.0212 (12)	0.0047 (12)	0.0047 (11)
C10	0.0479 (16)	0.0593 (14)	0.0470 (16)	0.0263 (13)	0.0054 (13)	0.0188 (13)
C11	0.0471 (15)	0.0400 (12)	0.0432 (15)	0.0208 (11)	−0.0017 (12)	0.0039 (11)
C12	0.0445 (15)	0.0424 (12)	0.0434 (15)	0.0193 (12)	−0.0055 (12)	−0.0001 (11)
C13	0.0531 (17)	0.0679 (16)	0.0442 (16)	0.0328 (14)	0.0044 (13)	0.0102 (13)
C14	0.0512 (17)	0.0751 (18)	0.0528 (18)	0.0372 (15)	−0.0016 (14)	0.0013 (15)
C15	0.0609 (19)	0.0463 (14)	0.0535 (18)	0.0282 (14)	−0.0088 (14)	0.0016 (12)
C16	0.066 (2)	0.0637 (17)	0.0558 (19)	0.0232 (16)	−0.0040 (16)	0.0218 (15)
C17	0.0493 (17)	0.0652 (17)	0.0586 (19)	0.0193 (14)	0.0005 (15)	0.0217 (15)
C18	0.072 (2)	0.0674 (17)	0.075 (2)	0.0387 (17)	−0.0184 (18)	0.0059 (17)
N1	0.0425 (13)	0.0618 (12)	0.0509 (14)	0.0255 (11)	0.0078 (11)	0.0217 (11)
N2	0.0397 (12)	0.0514 (11)	0.0443 (13)	0.0218 (10)	0.0059 (10)	0.0141 (10)
N3	0.0427 (12)	0.0599 (12)	0.0424 (13)	0.0252 (11)	0.0050 (10)	0.0107 (10)

### Geometric parameters (Å, º)

**Table:**

Cl1—C2	1.729 (3)	C10—C11	1.365 (4)
Cl2—C4	1.734 (3)	C10—N2	1.373 (3)
S1—C9	1.743 (3)	C10—H10	0.9300
S1—C8	1.753 (3)	C11—N3	1.398 (3)
O1—C1	1.356 (3)	C11—C12	1.465 (4)
O1—C7	1.413 (3)	C12—C13	1.381 (4)
C1—C6	1.388 (4)	C12—C17	1.390 (4)
C1—C2	1.395 (4)	C13—C14	1.379 (4)
C2—C3	1.374 (4)	C13—H13	0.9300
C3—C4	1.384 (4)	C14—C15	1.384 (4)
C3—H3	0.9300	C14—H14	0.9300
C4—C5	1.373 (4)	C15—C16	1.388 (4)
C5—C6	1.378 (4)	C15—C18	1.499 (4)
C5—H5	0.9300	C16—C17	1.386 (4)
C6—H6	0.9300	C16—H16	0.9300
C7—C8	1.487 (4)	C17—H17	0.9300
C7—H7A	0.9700	C18—H18A	0.9600
C7—H7B	0.9700	C18—H18B	0.9600
C8—N1	1.284 (3)	C18—H18C	0.9600
C9—N3	1.305 (3)	N1—N2	1.367 (3)
C9—N2	1.364 (3)		
			
C9—S1—C8	88.01 (12)	N2—C10—H10	127.3
C1—O1—C7	117.4 (2)	C10—C11—N3	110.8 (2)
O1—C1—C6	125.4 (2)	C10—C11—C12	126.9 (3)
O1—C1—C2	116.3 (2)	N3—C11—C12	122.2 (2)
C6—C1—C2	118.3 (3)	C13—C12—C17	117.1 (3)
C3—C2—C1	121.5 (2)	C13—C12—C11	122.4 (3)
C3—C2—Cl1	119.7 (2)	C17—C12—C11	120.5 (2)
C1—C2—Cl1	118.7 (2)	C14—C13—C12	121.3 (3)
C2—C3—C4	119.2 (3)	C14—C13—H13	119.3
C2—C3—H3	120.4	C12—C13—H13	119.3
C4—C3—H3	120.4	C13—C14—C15	122.0 (3)
C5—C4—C3	120.1 (3)	C13—C14—H14	119.0
C5—C4—Cl2	120.3 (2)	C15—C14—H14	119.0
C3—C4—Cl2	119.6 (2)	C14—C15—C16	116.8 (3)
C4—C5—C6	120.7 (2)	C14—C15—C18	121.9 (3)
C4—C5—H5	119.6	C16—C15—C18	121.3 (3)
C6—C5—H5	119.6	C17—C16—C15	121.2 (3)
C5—C6—C1	120.2 (3)	C17—C16—H16	119.4
C5—C6—H6	119.9	C15—C16—H16	119.4
C1—C6—H6	119.9	C16—C17—C12	121.5 (3)
O1—C7—C8	106.6 (2)	C16—C17—H17	119.3
O1—C7—H7A	110.4	C12—C17—H17	119.3
C8—C7—H7A	110.4	C15—C18—H18A	109.5
O1—C7—H7B	110.4	C15—C18—H18B	109.5
C8—C7—H7B	110.4	H18A—C18—H18B	109.5
H7A—C7—H7B	108.6	C15—C18—H18C	109.5
N1—C8—C7	121.1 (2)	H18A—C18—H18C	109.5
N1—C8—S1	116.9 (2)	H18B—C18—H18C	109.5
C7—C8—S1	121.93 (19)	C8—N1—N2	108.3 (2)
N3—C9—N2	113.1 (2)	C9—N2—N1	118.9 (2)
N3—C9—S1	139.0 (2)	C9—N2—C10	106.8 (2)
N2—C9—S1	107.86 (19)	N1—N2—C10	134.3 (2)
C11—C10—N2	105.4 (2)	C9—N3—C11	103.8 (2)
C11—C10—H10	127.3		
			
C7—O1—C1—C6	12.1 (4)	C10—C11—C12—C17	3.8 (4)
C7—O1—C1—C2	−168.5 (2)	N3—C11—C12—C17	−176.8 (2)
O1—C1—C2—C3	179.8 (2)	C17—C12—C13—C14	0.3 (4)
C6—C1—C2—C3	−0.8 (4)	C11—C12—C13—C14	−179.7 (2)
O1—C1—C2—Cl1	0.1 (3)	C12—C13—C14—C15	0.7 (4)
C6—C1—C2—Cl1	179.52 (18)	C13—C14—C15—C16	−1.2 (4)
C1—C2—C3—C4	0.4 (4)	C13—C14—C15—C18	179.8 (3)
Cl1—C2—C3—C4	−179.96 (18)	C14—C15—C16—C17	0.6 (4)
C2—C3—C4—C5	0.4 (4)	C18—C15—C16—C17	179.7 (3)
C2—C3—C4—Cl2	−178.67 (19)	C15—C16—C17—C12	0.4 (4)
C3—C4—C5—C6	−0.7 (4)	C13—C12—C17—C16	−0.8 (4)
Cl2—C4—C5—C6	178.4 (2)	C11—C12—C17—C16	179.2 (3)
C4—C5—C6—C1	0.2 (4)	C7—C8—N1—N2	−178.7 (2)
O1—C1—C6—C5	179.9 (2)	S1—C8—N1—N2	0.5 (3)
C2—C1—C6—C5	0.5 (4)	N3—C9—N2—N1	179.9 (2)
C1—O1—C7—C8	173.7 (2)	S1—C9—N2—N1	0.0 (3)
O1—C7—C8—N1	175.6 (2)	N3—C9—N2—C10	0.9 (3)
O1—C7—C8—S1	−3.6 (3)	S1—C9—N2—C10	−178.94 (16)
C9—S1—C8—N1	−0.4 (2)	C8—N1—N2—C9	−0.3 (3)
C9—S1—C8—C7	178.8 (2)	C8—N1—N2—C10	178.3 (3)
C8—S1—C9—N3	−179.7 (3)	C11—C10—N2—C9	−0.6 (3)
C8—S1—C9—N2	0.19 (17)	C11—C10—N2—N1	−179.3 (2)
N2—C10—C11—N3	0.1 (3)	N2—C9—N3—C11	−0.8 (3)
N2—C10—C11—C12	179.6 (2)	S1—C9—N3—C11	179.0 (2)
C10—C11—C12—C13	−176.2 (2)	C10—C11—N3—C9	0.4 (3)
N3—C11—C12—C13	3.2 (3)	C12—C11—N3—C9	−179.1 (2)
